# A standard for test reliability in group research

**DOI:** 10.3758/s13428-012-0223-z

**Published:** 2012-06-27

**Authors:** Jules L. Ellis

**Affiliations:** School of Psychology and Artificial Intelligence, Radboud University Nijmegen, P.O. Box 9104, 6500 HE Nijmegen, The Netherlands

**Keywords:** Attenuation, Coefficient alpha, Efficient, Reliability, Power

## Abstract

Many authors adhere to the rule that test reliabilities should be at least .70 or .80 in group research. This article introduces a new standard according to which reliabilities can be evaluated. This standard is based on the costs or time of the experiment and of administering the test. For example, if test administration costs are 7 % of the total experimental costs, the *efficient* value of the reliability is .93. If the actual reliability of a test is equal to this efficient reliability, the test size maximizes the statistical power of the experiment, given the costs. As a standard in experimental research, it is proposed that the reliability of the dependent variable be close to the efficient reliability. Adhering to this standard will enhance the statistical power and reduce the costs of experiments.

Reliabilities are routinely reported in group research in which psychological tests or questionnaires are used. But how high should reliabilities be? Throughout this article, the word *standard* is used to refer to a normative rule that specifies values that are considered optimal or acceptable. In the past century, considerable progress has been made in defining and estimating reliability coefficients (Biemer, Christ, & Wiesen, 2009; Brennan, 2001; Cronbach & Shavelson, 2004; Sijtsma, 2009), but virtually no attention has been given to the development of standards of reliability. The absence of such standards is a serious gap in existing behavioral research methods and leads one to ask why reliabilities are routinely computed if their acceptable values are unknown.

This article introduces a rational standard for test reliability. It is applicable in research in which differences between group means are studied and test scores are used as the dependent variable, and in which the main goal is to decide whether the null hypothesis of equal group means is true. This article does not discuss reliability standards for individual decisions, because several authors (e.g., Nunnally & Bernstein, 1994) have argued that different standards should be applied for tests that are used in group research versus those used for individual decisions.

One assumption of this article is that the researcher wants to design the study such that it maximizes the statistical power of the *t* test or ANOVA that is used to compare the group means. It will be assumed that Group is a fixed factor. Recall that, in a *t* test or ANOVA, the researcher sets the level of significance in advance, usually at 5 %. In the past decades, it has increasingly been recognized that it is also important that the statistical power be high. A high power is needed because, otherwise, the experiment will too easily yield nonsignificant results, due to a lack of observations (see Cohen, 1988, for more information). The main method to increase power is to increase the number of observations.

A second assumption of this article is that the researcher wants to minimize the costs of the study, where costs are measured in either money or participant time. This constrains the number of observations and, hence, the power. Therefore, these two wishes, maximizing power and minimizing costs, conflict with each other. Two ways to reconcile them are (1) to maximize the power, under the restriction that the costs are fixed at a given budget; or (2) to minimize the costs, under the restriction that the power is fixed at a given target. Both goals are equivalent for the purpose of this article.

Two types of sample size are included in this design: the number of subjects and the number of measurements (e.g., test items, raters, or time points). Their effects on power are summarized in Fig. 1. In a traditional power analysis, one would consider the number of subjects, the difference of the means, and the standard deviation. In the present situation, increasing the number of measurements will increase the reliability of the test, and thus decrease the standard deviation and increase the power. Increasing the number of subjects will also increase the power. Now, consider the goal of maximizing the power under the restriction that the costs are fixed. For fixed costs, one has to allocate one part of the budget to the recruitment of subjects and another part to test administration (e.g., paying the raters). Thus, there are two sample sizes, and both increase the power, but if one sample size increases, then the other must decrease—otherwise, the budget will be exceeded. So, the question is which allocation rule maximizes the power of a *t* test, given the budget.

**Fig. 1:**
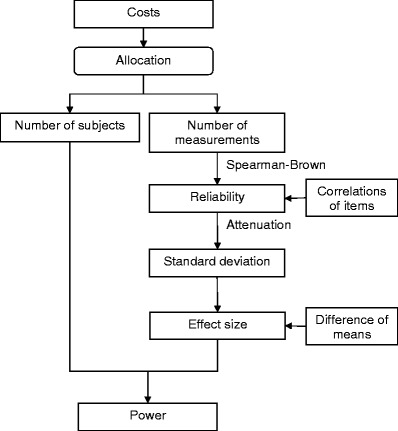
Relationships between major concepts that mediate between the cost and power of an experiment

As will be discussed, this goal—to maximize power for a fixed budget—implies a rule for the reliability of the test by which the dependent variable is measured. By this rule, researchers can judge whether the test is efficient for the experiment. Thus, instead of simply comparing the test’s reliability with .70 or .80, the reliability will be compared with a norm that is specific for the experiment and its costs. This norm will be called the *efficient reliability*. If the actual reliability is smaller than the efficient reliability, the power can be increased with a fixed budget by increasing the reliability—that is, using more measurements with fewer subjects. If the actual reliability is larger than the efficient reliability, the power can be increased with the same budget by decreasing the reliability—that is, using fewer measurements with more subjects.

This article is organized as follows: The argument that there currently is no convincing standard for test reliability in group research is presented, and a summary is provided of the effects of reliability on the outcomes of group research. The effect of test size on reliability is summarized, and it is argued that a reliability standard can be based on the value of reliability that maximizes the efficiency or the power of a design. Finally, the question of how this concept can be used in test construction and group research is addressed.

## Current reliability standards

Authors who wish to determine whether their computed reliability is sufficiently high often cite Nunnally (1978, p. 245; Nunnally & Bernstein, 1994), who stated that “increasing reliabilities much beyond .80 is often wasteful of time and funds” in basic research, because such an increase has little effect on correlations. This argument involves the balance between the cost of improving reliability and the effect of increased reliability on the outcomes of a study. However, because costs vary between research fields, it may be impossible to set a general standard for reliability across fields.

Another frequently used source of reliability standards is common practice. In a discussion of coefficient alpha in applied psychological research, Cortina (1993) remarked:

One reason for the misuse of alpha in applied psychology is that there seems to be no real metric for judging the adequacy of the statistic. Experience with the literature gives one some general idea of what an acceptable alpha is, but there is usually little else to go on. (p. 101)

Similar remarks could be made with respect to standards for effect sizes. The common-practice standard is often used implicitly, as indicated by Cortina (p. 101): Alphas larger than .70 are usually presented without comment and without further attempts to improve the scale. Similarly, Frisbie (1988, p. 29) stated that some standards are based on the observed reliabilities of tests that are regarded as acceptable by most. However, a standard cannot be derived simply on the basis of common practice, because such reasoning is circular.

Many psychometric books devote no more than a few paragraphs to reliability standards and use rather intuitive arguments. Authors may use a combination of the arguments above, may not specify a standard value, or may avoid discussing the problem altogether. Although the most obvious source for such standards would be the *Standards for Educational and Psychological Testing* (American Educational Research Association, American Psychological Association, & National Council of Measurement in Education, 1999), this text does not specify standards for reliability values.

Some authors seem to consider alpha as a measure of the internal consistency of the test. Although alpha may be called an “internal consistency reliability,” this name reflects that alpha is *influenced* by—among other things—internal consistency, not that it is a *measure* of internal consistency. The interpretation of alpha as a measure of internal consistency has been criticized repeatedly for decades—for instance, by (Sijtsma 2009, p. 119). The main problem of interpreting alpha as a measure of internal consistency is that alpha tends to increase with the number of items, a problem that was already discussed by (Cronbach 1951, p. 323). For example, consider a test consisting of two uncorrelated factors, with 50 % of the items loading on Factor 1 but not on Factor 2, and 50 % of the items loading on Factor 2 but not on Factor 1. Assume, for simplicity, that all nonzero loadings are 0.6 and that all items have equal variances. With four items, alpha will be .35, and with 20 items alpha will be .80. But the only difference is that more items of the same kind have been added; the items did not become more consistent. Interpreting alpha as a measure of internal consistency is comparable to confusing significance and effect size, thus ignoring the effect of sample size. It will be assumed here that if alpha is reported, it is reported only as an estimate of reliability. Note, furthermore, that this article is about reliability, not necessarily about alpha.

## Effect of reliability in group research

Essential to the arguments of Nunnally (1978) and others is the effect of reliability on correlations. This effect is described by the attenuation formula (Spearman, 1904; see also Lord & Novick, 1968),

$$ {\rho_{{XY}}} = {\rho_{{T(X)T(Y)}}}\sqrt {{{\rho_X}{\rho_Y}}}, $$

where *ρ*_*XY*_ is the correlation between manifest variables *X* and *Y*, *ρ*_*T*(*X*)*T*(*Y*)_ is the correlation between their true scores, and *ρ*_*X*_ and *ρ*_*Y*_ are their reliabilities. A similar relationship holds in a two-group design for the (standardized) effect size, Cohen’s *d*. If the true, error-free value of the effect size is denoted by *δ*_*T*(*Y*)_ and the population value of the effect size of the manifest variable is denoted by *δ*_*Y*_, then

$$ {\delta_Y} = {\delta_{{T(Y)}}}\sqrt {{{\rho_Y}}} $$

(Cohen, 1988, p. 536). Such a formula is also applicable for the influence of reliability on the effect size in an analysis of variance (ANOVA) for a balanced design (Cohen, 1988; Feldt, 2011).

These formulas indicate that increasing the reliability beyond .80 will have only a limited effect on the outcome of the research. However, this observation does not justify the conventional standard of at least .70 or .80 for reliabilities in group research. Consider a one-sided significance test of the null hypothesis, *ρ*_*XY*_ = 0 or *δ*_*Y*_ = 0. When the reliability value is positive, it is possible to compensate for the attenuation by adding more subjects. A test with a significance level of .05, power of .80, and *δ*_*T*(*Y*)_ = 0.80 would require *N* = 42 if *ρ*_*Y*_ = 1. However, if *ρ*_*Y*_ = .10, then the same significance level and power could be achieved with *N* = 388. Thus, for the purpose of significance testing, any positive reliability is high enough, provided that enough subjects can be added to the research.

Additionally, consider the estimation of effect size. With a reliability of .10, the true effect size, *ρ*_*T*(*X*)*T*(*Y*)_ or *δ*_*T*(*Y*)_, would be seriously underestimated if one believes it to be equal to the observed effect size, *ρ*_*XY*_ or *δ*_*Y*_. However, if the reliability is known, then one can correct for attenuation (Charles, 2005; Mendoza, Stafford, & Stauffer, 2000). In its simplest form, this correction means that the observed effect size is divided by the square root of the reliability. For example, if the observed effect size is 0.40 and the reliability is .25, then the researcher can compute the true effect size as ${{{0.40}} \left/ {{\sqrt {{.25}} }} \right.} = 0.80$. The essential condition for this correction is that the reliability is known, not that it is high.

Whether correction for attenuation is appropriate has been debated for decades (see, e.g., Borsboom & Mellenberg, 2002; Charles, 2005; DeShon, 1998; Schmidt & Hunter, 1999). Only two points of this debate will be addressed here. A practical complication of the correction for attenuation is that many researchers use coefficient alpha to estimate reliability. There are two problems with this. First, there can be discussion about which form of reliability is more appropriate: internal consistency reliability, interrater reliability, or test–retest reliability. I will deliberately avoid taking a position about this in the present article, because the conclusions here are applicable to all three reliability forms, and there is no need to antagonize readers who have a strong opinion about this.

Second, if internal consistency reliability is being used, alpha is generally only a lower bound of the reliability (Lord & Novick, 1968), and it can lead to overestimation of the true score correlation if it is used in a correction for attenuation. Alpha was first introduced more than 60 years ago (Guttman, 1945), and more generally applicable estimates of reliability have been developed since (Bentler, 2009; Green & Yang, 2009; Osburn, 2000; Rae, 2007; Revelle & Zinbarg, 2009; Sijtsma, 2009; ten Berge & Sočan, 2004). Nicewander (1990) derived an upper bound for the reliability of binary items that does not overestimate the true score correlation when used in the correction for attenuation. Furthermore, under various models (e.g., if the items are essentially tau-equivalent), one can obtain an accurate estimate of the reliability. (In this article, the term *accurate estimate* will be used synonymously with the statistical term *consistent estimator*, which is an estimator that converges to the correct value as the sample size goes to infinity).

The conclusion is that attenuation formulas do not justify the standard that reliabilities in group research should be at least .80. Instead, it is also sufficient that the reliability value be positive if null-hypothesis testing is the main objective. For effect size estimation, it is sufficient that correction for attenuation may be applied. This requires that the reliability value be positive and be estimated accurately (which may require a valid measurement model and other estimates than alpha). In research in which effect size estimation is the main objective but correction for attenuation is not trusted, it may be justified to require that the reliability be at least .80, but that does not the preclude the possibility, shown in the analysis below, that an even higher reliability may be needed to make the design efficient.

## Increasing reliability by adding components

It will be assumed henceforth that, in theory, the researcher has the possibility of increasing the reliability by adding more measurement components to the test. That is,

- If internal consistency reliability is used, the researcher can add more items;
- If interrater reliability is used, the researcher can add more raters;
- If test–retest reliability is used, the researcher can add more retests.

It is *not* assumed that these additional components are actually available; it is sufficient that, in theory, they can be added. For example, suppose that a panel of two students is trained to judge the aggressiveness of young children. After applying the method of this article, the conclusion may be that more raters are needed in order to achieve efficient reliability. The additional raters do not exist yet, because the researcher trained only two students. But, clearly, the researcher could train more students; in this sense, the additional raters exist “in theory,” and it is meaningful to consider what the reliability would be if they were added to the panel. This is different if the raters have to be the parents of the child. In that case, it is not possible to add more similar raters, and it is futile to compute what the reliability would be if the researcher used more parents. Similarly, it is assumed that the researcher can add similar items to a test, even if such items have not been constructed yet.

If components are added to the test, the total score over all components would be used as a dependent variable. The relevant reliability is the reliability of this total score. This reliability can be estimated in various ways. One possible estimate is the intraclass correlation for consistency of average measurements [*ICC*(*C*,*k*) from McGraw & Wong, 1996], which is equal to coefficient alpha when the components are items. More sophisticated estimates can be obtained on the basis of latent variable models or multilevel analysis.

If components are added to the test, the reliability of the total score usually increases. It will be assumed here that, if the number of components is increased by a factor of *k*, the reliability increases according to the Spearman–Brown formula (e.g., Lord & Novick, 1968). That is, if *ρ* is the reliability of the current test and *ρ*_*k*_ is the reliability of the new test, then

$$ {\rho_k} = \frac{{k\rho }}{{1 + \left( {k - 1} \right)\rho }} $$

A sufficient condition for this is that the components be essentially tau-equivalent with equal error variances, a condition that may be be called *essentially parallel*. However, the Spearman–Brown formula can also be valid in other circumstances. The Spearman–Brown formula also holds for the reliability of a weighted sum if the components are congeneric with equal reliabilities (Drewes, 2000). Furthermore, if a test consists of 15 components and is lengthened by a factor of 2, it is not necessary that all 30 components be essentially parallel with each other; it is sufficient that the sum of the 15 old components be essentially parallel with the sum of the 15 new components. For a large number of components that are sampled randomly from a domain, it may be expected that tests of equal length will be approximately parallel, so in that case the Spearman–Brown formula can still serve as an educated guess for large changes in test length.

One could object that the Spearman–Brown formula requires certain assumptions, as described above, that are hard to fulfill. But, for the derivation below, it is sufficient that such components can exist; it is not needed that they actually be available. This is comparable to a statistician who may theorize about the potential effect of increasing the sample size, even if the size of a specific sample is not increased in reality.

## Reliability and efficiency

Although a low reliability can be compensated for by the inclusion of a large number of subjects, this approach is not necessarily efficient. If it is costly to obtain subjects and inexpensive to add new measurement components (items, raters, or retests, depending on the reliability form that is appropriate), then it is more efficient to add new components rather than new subjects. Hence, the goal is to find the right balance between subjects and components.

The various test components can be considered as repeated measures. Many authors have studied cost optimization in designs with repeated measures (Allison, Allison, Faith, Paultre, and Pi-Sunyer, 1997, p. 27, and references therein). Here, the same reasoning is applied to test reliability. Suppose that there is a fixed cost associated with each subject, due to the processes of selecting the subject, reading the instructions, and conducting the experimental manipulations. Moreover, assume that there is an additional cost for each component (item, rater, or retest) per subject. Suppose that the fixed cost per subject is *b* and that the cost of administering an existing test to one subject is *c*. Without loss of generality, it can be assumed that the unit of measurement of the costs is chosen such that *b* + *c* = 1; then *c* is also the *proportion* of the costs of administering the test. If the number of components of the existing test is increased by a factor of *k*, the costs of a study with *n* subjects would be

$$ n\left( {b + ck} \right) $$

The noncentrality parameter of a *t* test for independent samples with equal sample sizes would be

$$ {\lambda_k} = {\delta_{{T(Y)}}}\sqrt {{{\rho_k}{{n} \left/ {2} \right.}}} $$

This parameter determines the power of the *t* test. The power would also be influenced by the degrees of freedom, but that effect becomes negligible when *n* is large. Thus, when the researcher sets a power goal, this choice is practically equivalent to choosing a value of *ρ*_*k*_ *n* that must be achieved by making *k* and *n* sufficiently large. Next, the costs $n\left( {b + ck} \right)$ must be minimized under the restriction that *ρ*_*k*_ *n* is constant. The method of Lagrange multipliers is used to identify the minimum at

$$ k = \sqrt {{\frac{{\left( {1 - \rho } \right)}}{\rho }\frac{b}{c}}} $$

(see, e.g., Allison et al., 1997). An alternative derivation is this: Assume a fixed budget and maximize the power. The restriction of a fixed budget *B* implies that the number of subjects is a decreasing function of the number of components: $n = {{B} \left/ {{\left( {b + ck} \right)}} \right.}$. Next, substitute this and the Spearman–Brown formula in the formula of λ_*k*_, take the derivative of λ_*k*_ with respect to *k*, set the derivative equal to zero, and solve for *k*. This formula can also be derived for a balanced ANOVA, because its effect size satisfies a similar attenuation formula (Cohen, 1988; Feldt, 2011).

It was assumed that the unit of measurement for the costs is chosen such that *b* + *c* = 1. If the current test is already optimal, then the outcome must be *k* = 1. Substituting these two equalities in the formula for the optimal *k* yields $1 = \left( {1 - \rho } \right){{{\left( {1 - c} \right)}} \left/ {{\left( {\rho c} \right)}} \right.}$. This can be solved to create the equation

$$ \rho = 1 - c $$

This outcome, 1 – *c*, will henceforth be called the *efficient reliability* (*ρ*_eff_). For example, suppose that internal consistency reliability or test–retest reliability is being used, and that the costs are proportional to the time that a subject spends on the study. Suppose that an experiment of 1 h total length is conducted, of which 10 min is reserved for administration of the test. This 10 min corresponds to 17 % of the costs. Therefore, the efficient reliability is 1 – .17 = .83. Similarly, if interrater reliability is being used and the raters are 17 % of the costs, the efficient reliability is likewise .83.

**Fig. 2:**
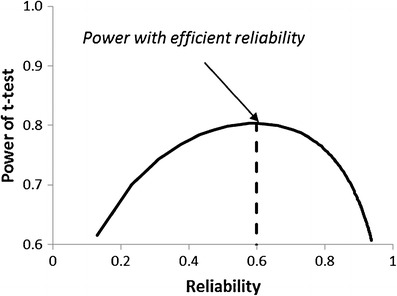
Power of a *t* test as function of reliability, in an experiment with fixed budget and for a test with current reliability = efficient reliability = .6 when the true effect size *δ* = 0.5 and current *n* = 50

An example of the effect of reliability on the power of a *t* test in an experiment with fixed budget is shown in Fig. 2. In this example, the reliability of the current test is .60, and the efficient reliability is also .60. It is assumed that the true effect size is 0.50 and that the budget allows for 50 subjects with the current test. The figure shows how the power changes as a function of reliability when the test length is changed. For the current test length, the power is .80. If the test is shortened, the reliability decreases and then the power decreases. But if the test is lengthened, the power decreases too: If the reliability is increased to .90, the power decreases from .80 to .66. The reason is that, in order to get a reliability of .90, the test length must be increased by a factor of 6, but then the budget allows for only 16 subjects instead of the original 50. The negative effect of the decreasing number of subjects, forced by a fixed budget, outweighs the positive effect of the increasing reliability.

With respect to both internal consistency reliability and test–retest reliability, the total time needed for testing may be assumed to be proportional to the number of components (items or retests, respectively). Assuming that participant time is equivalent to costs, one reaches the following conclusion: For long experiments, in which the test time is a small fraction of the total experiment time, a high reliability is needed. However, in short experiments and survey studies, if the test time is a relatively large fraction of the total time, it makes sense to accept the low reliability of a short test and use the saved money to recruit more subjects. A similar conclusion is possible for interrater reliability if “time” in the analysis above is replaced by “costs.” Three examples from the literature are given below to illustrate this relationship between reliability and study cost. These examples use participant time as the cost measure.

Sørensen, Birket-Smith, Wattar, Buemann, and Salkovskis (2011) conducted a study in which patients received 16 sessions of approximately 60 min of psychotherapy. Several questionnaires, including the 14-item Health Anxiety Inventory, were used to measure the dependent variables. Suppose that answering the questionnaire had cost 30 min per patient (the actual time was not reported by Sørensen et al.). This 30 min represents 3 % of the total time, which leads to an efficient reliability of 1 – .03 = .97. If the therapist’s time instead of the patient’s time is used as the cost measure, the efficient reliability would be lower, because some therapy sessions were group sessions.

Nisbet and Zelenski (2011) conducted a study in which students took a 17-min walk, indoors or outdoors. The Positive and Negative Affect Schedule (PANAS) was used “to assess positive and negative affect, each with a 10-word list of emotions, (p. 1102)” and *t* tests were conducted to compare the indoor walk with the outdoor walk. Watson and Clark (1999) have stated that most subjects complete the expanded, 60-item PANAS-X in 10 min or less. So, it seems reasonable to assume that most subjects in the Nisbet and Zelenski study completed the 10-item Positive Affect scale within approximately 1.7 min. Its efficient reliability for this experiment is therefore 17/(17 + 1.7) = .91. This computation ignores that subjects also completed “demographic and filler questionnaires,” for which no time was reported; if this took 10 min per subject, the efficient reliability would be .94. The time needed to complete the second PANAS scale was not included in this computation.

Lewis, Granato, Blayney, Lostutter, and Kilmer (2011) conducted a Web-based survey on sexual behavior and alcohol use. Their Table 1 contains results from *t* tests for the differences between men and women on six dependent variables. These dependent variables were measured by one, one, four, five, five, and five items, respectively. One of the dependent variables was the score on a five-item positive affect scale with respect to the most recent sexual encounter, which will serve as the focus for the present example. In theory, the other scales can be shortened or lengthened, too; therefore, it will be assumed that the fixed part of the study consists of one item per variable. The results of Lewis et al.’s Table 1 are thus based on at least six questions (one for sex and five for the other dependent variables), apart from the five-item target variable. Assuming that each question requires the same amount of time to answer, the efficient reliability of the positive affect scale would be 6/(5 + 6) = .55. The present example is limited to Lewis et al.’s Table 1 in order to illustrate that the efficient reliability can be lower than .70 in principle. In reality, however, Lewis et al. included more questions and more analyses.

Note that different concepts of cost can be utilized. Allison et al. (1997) discussed an elaborate example in which costs were expressed in dollars. In the examples above, participant time was used as the cost measure. Even if participants are not being paid, it may be ethical to minimize the total time load involved in participating. Using participant time also has the advantage that it is probably more comparable for studies with different participant remuneration and cost structures. Participant time is not the appropriate cost measure if interrater reliability is used and the raters work simultaneously, however.

Application of the formula can be reversed if participant time is used as the cost measure. Given the time needed to take the test and its reliability, one can compute the total experimental length for which the given reliability is efficient. For example, suppose that a 15-min test has a reliability of .80; this reliability is efficient if the total experimental time is 75 min. For shorter experiments, the test is more reliable than necessary; for longer experiments, a longer test (more items or more retests, depending on the reliability form that is appropriate) would be needed to achieve efficient reliability.

Although it is possible to change the internal consistency reliability by altering the length of a test, I do not recommend that researchers routinely change existing tests. This argument was merely used as a theoretical possibility to derive the efficient reliability. In reality, it is usually important that the results be comparable to those of other studies and that an instrument that has been validated previously be maintained; therefore, repeatedly changing tests would be inappropriate. Nevertheless, when researchers do determine that changing a test is warranted, they should be aware that altering test length will also change the efficient reliability.

Consider, for example, a scenario in which the current internal consistency reliability is *ρ* = .50 and *c* = .20. In this case, the efficient reliability is *ρ*_eff_ = .80. The fact that *ρ* < *ρ*_eff_ means that, for these costs, one would want a more reliable test. If the test were lengthened, it would take a greater proportion of the total costs, and the efficient reliability would become smaller. In this example, the values of *ρ* and *c*, when substituted in the formula for the optimal *k*, yield *k* = 2. Substituting this *k* and *ρ* into the Spearman–Brown formula yields a value of .67, which may be called the *reliability goal*. Although it could be argued that this reliability goal should be considered as the efficient reliability value (rather than *ρ*_eff_), the ensuing formula is considerably more complicated than the conceptually simple definition of efficient reliability given above. Furthermore, the reliability goal is related almost linearly to *ρ*_eff_ for *ρ*_eff_ values between .10 and .90 (*r* = .998). Therefore, I conclude that the definition of efficient reliability, *ρ*_eff_, is the more useful formula in practice.

## Discussion

*As a standard of reliability in group research, it is proposed that the reliability of the dependent variable be close to the efficient reliability*. Thus, in designing experiments, if different test versions with different reliabilities exist, researchers may select the version for which the reliability is closest to the efficient reliability in the planned experiment. In designing new tests, test constructors may consider the kinds of experiments in which the test will be used and may aim for a reliability that is close to the efficient reliability in these experiments. In evaluating existing experiments and tests, readers may compare the reported reliability with the efficient reliability and, if there is a large difference, ask why the researchers did not use a more efficient number of items, raters, or retests.

Several possible misconceptions should be addressed here. It is not claimed that the standard proposed above is exhaustive. Additional standards may be warranted, and these may conflict with the one stated above. For example, if the researcher plans other analyses besides *t* tests and ANOVAs, these analyses may require different reliabilities. In such cases, the concept of efficient reliability will still be useful as one of the optima. For example, suppose that the efficient reliability is .90 and a second analysis requires a reliability of 1. Then, every value between .90 and 1 is defensible as a compromise between the two optima, while the classical standard of .70 does not have a rational defense. An example of this situation would be if a researcher wants to assess the unidimensionality of the test. Demonstrating unidimensionality can contribute to the construct validity of the test, but it can be problematic if the internal consistency reliability is low. If unidimensionality is assessed by a factor model (e.g., Wirth & Edwards, 2007), then correct recovery of the population factors will depend on the degree of overdetermination of the factors (MacCallum, Widaman, Zhang, & Hong, 1999), which is related to the internal consistency reliabilities of the subtests corresponding to the factors.

A second example of additional standards is this: If the efficient reliability is low, say .30, a researcher may hesitate to implement the experiment with a test that has this reliability, because this would make it difficult for other researchers to replicate the findings with a smaller subject sample. Here, the second standard is “ease of replication.” But note that several examples have had an efficient reliability above .90. In those cases, it would be hard to defend the conventional standard of .70, since both efficiency and ease of replication would benefit from a higher reliability. Thus, even if one does not allow reliabilities below .70, it is still useful to compute the efficient reliability.

Another reason why one may hesitate to allow reliabilities much smaller than .70 is that these would be inadequate for calculating confidence intervals around individual scores and individual diagnostic decisions. As was stated in the introduction, the standard of efficient reliability is applicable only in certain kinds of group research, and not for individual decisions.

Although a low reliability can be compensated for by adding more subjects to the research, a lack of construct validity cannot be compensated for in that way. But increasing the reliability by adding similar items, raters, or retests does not improve construct validity, either. Reliability is relevant only if the construct validity of the test is assumed to be adequate first; hence, the same is true for the reliability standard.

If the internal consistency reliability or the interrater reliability of a test is efficient for a given experiment, this does not imply that the quality of the items or raters is optimal. Indeed, the same test can be inefficient for another experiment. Achieving the efficient reliability means that the *number* of items or raters is efficient for the experiment, given the quality of the items or raters.

I do not advise that researchers routinely change the length of preexisting tests; there are obvious advantages of using a standard test. However, once the validity and internal consistency reliability of a newly constructed test have been established in the initial construction phase, it may be worthwhile to develop shorter versions to accommodate the various reliability needs that exist in group research. This process is already sometimes performed, although usually with a minimum alpha of .70. According to the present analysis, it may also be useful to develop versions with a lower reliability, such as .30. Moreover, researchers who choose a brief test version in order to minimize the time load per subject should be aware that they might not minimize the total time load over all subjects.

Finally, the proposed standard should be applied with leniency. For example, suppose that the efficient reliability of a test is .95 and the actual reliability is .90. Although this design may not be the most efficient, it seems unreasonable to reject the study for that reason alone. However, if the actual reliability is .70, it seems fair to ask why the researchers did not use a more reliable test. If they attempted to comply with a standard—any standard, for that matter—and failed, there might be a problem with the validity of the test.

In conclusion, the concept of efficient reliability developed in this article can be used to evaluate the reliability of a test in a given experiment. The best reliability is not always 1 or .80. Low reliabilities are associated with cheap tests, which can maximize the power of a study while keeping the costs fixed if the subjects are relatively inexpensive. On the other hand, reliabilities well above .90 can be desirable in experiments that require large investments per subject.

This article has focused on a simple design to illuminate the principle that a rational reliability standard is possible. Similar analyses of other research designs may contribute to the development of rational reliability standards in the future. For example, a different, but equally simple, formula can be derived for the efficient value of internal consistency reliability in situations in which the number of subjects decreases exponentially with the number of items. In that case, the reliability that maximizes the power is 1 – λ, where λ is the exponential decay rate. This formula may be applicable in Web surveys. Further research may extend the theory to multivariate research, nonparametric tests, and multilevel analysis.
